# Resolution of generalized tonic seizures following focal ablative or resective surgery

**DOI:** 10.1002/epd2.70148

**Published:** 2026-01-19

**Authors:** Sem L. Kampman, Itay Tokatly Latzer, Aaron E. L. Warren, Jurriaan M. Peters

**Affiliations:** ^1^ Localization Laboratory, Division of Epilepsy and Clinical Neurophysiology, Department of Neurology, Boston Children's Hospital Harvard Medical School Boston Massachusetts USA; ^2^ Faculty of Medicine, University Medical Center Utrecht Utrecht University Utrecht The Netherlands; ^3^ Gray School of Medicine, Gray Faculty of Medical and Health Sciences Tel‐Aviv University Tel Aviv Israel; ^4^ Department of Neurosurgery, Mass General Brigham Harvard Medical School Boston Massachusetts USA

**Keywords:** brain networks, cortical lesions, LITT, pediatric epilepsy, resective surgery, tonic seizures

## Abstract

**Objective:**

Focal brain lesions may underlie generalized tonic seizures, as seen in Lennox–Gastaut syndrome, by engaging bilateral neural networks. However, this seizure type is often not considered surgically remediable. Here, we describe the resolution of apparent electroclinically classic generalized tonic seizures in children originating from a unifocal brain lesion following resective or ablative surgery. This study aims to contribute to emerging evidence that prompt removal of a lesion may resolve generalized seizures by ameliorating aberrant network activity.

**Methods:**

Boston Children's Hospital's (BCH) epilepsy surgical database was reviewed to identify children with tonic seizures due to a focal brain lesion who remained seizure‐free for longer than 1 year following resective or ablative surgery.

**Results:**

Five children were identified, of whom three underwent resective surgery and two laser interstitial thermal therapy (LITT). Age at epilepsy onset varied from 1 month to 7.25 years, and age at first epilepsy surgery ranged from 5.6 to 9.5 years. Lesions were predominantly located in the frontal lobe (*n* = 3), and focal cortical dysplasia (FCD) was the most common underlying etiology (*n* = 3), followed by vascular lesions (*n* = 2). At last follow‐up, seizure freedom (Engel Class 1A) ranged between 1.7 and 4.4 years.

**Significance:**

This study presents evidence that resection or ablation of a focal cortical lesion can resolve generalized tonic seizures. The findings also lend credence to the hypothesis that heterogeneous brain lesions can underlie shared electroclinical features through engaging similar brain networks. Children with tonic seizures in whom a lesional etiology is presumed should undergo timely surgical evaluation, as removal of a focal lesion may arrest the evolution of a secondary epileptic network and allow for the restoration of normal brain network development.

**Key points**

- Heterogenous cortical lesions can underlie generalized tonic seizures through converging on shared brain networks.
- Surgical resection or ablation of a focal brain lesion can lead to sustained seizure freedom for generalized tonic seizures.
- Early surgical removal of a cortical lesion may impede the development of secondary epileptic networks and thereby restore normal brain network development.
- Patients with tonic seizures in whom a focal cause is confirmed or suspected should promptly be referred for surgical evaluation.

## 1 INTRODUCTION

Tonic seizures are the principal seizure type of Lennox–Gastaut syndrome (LGS), and often persist into adulthood.^1^, ^2^ A cortical–subcortical hierarchy has been postulated to underlie tonic seizures, where epileptic activity rapidly propagates from the frontal cortex to the brainstem's reticular formation via extrapyramidal corticoreticular pathways,^3^ with the thalamus involved secondarily. Tonic seizures are typically highly medically refractory.^2^, ^4^

When these seizures originate from diffuse structural, genetic, or other etiologies, and the seizure semiology is without localizing features, surgical management may be limited to disconnection procedures, including corpus callosotomy, and to neuromodulation techniques, including vagus nerve stimulation (VNS), deep brain stimulation (DBS), or responsive neurostimulation (RNS).^5^ In approximately 20% of cases, tonic seizures may be caused by focal structural abnormalities, such as focal cortical dysplasia (FCD) or hypoxic–ischemic injuries.^6^, ^7^ Such lesions can cause a generalized electroclinical phenotype through interacting with large‐scale brain networks.^6^, ^8^ In this scenario, resective or ablative surgery can reduce or even stop tonic seizures^5^, ^9^, ^10^ and may improve cognitive and behavioral outcomes in some patients.^9^, ^10^, ^11^, ^12^ Epilepsy surgery has also been successful in children with other forms of generalized ictal and interictal EEG patterns in children,^13^ including resective surgery and hemispherectomy for electrical status epilepticus of sleep (ESES) and hemispherectomy and (multi)lobar resection for slow spike–wave complexes.^13^, ^14^

Despite these potential benefits, epilepsy surgery remains underutilized in LGS and in patients with tonic seizures who do not meet all criteria for LGS.^15^, ^16^ Generalized seizures are often considered for nonresective or ablative procedures only, even when the cause could be focal. A potential barrier to surgery for tonic seizures may be an exaggerated emphasis on achieving complete seizure freedom,^17^ though evidence suggests that even modest reductions in seizure frequency can improve quality of life.^16^, ^18^ Additionally, nonseizure outcomes such as anxiety, cognition, and depression are likely important determinants of quality of life,^19^, ^20^ and these can also improve after epilepsy surgery.^9^, ^16^

Here, we present a case series of children with apparent electroclinically generalized tonic seizures stemming from a focal structural abnormality, in whom resective epilepsy surgery or laser interstitial thermal therapy (LITT) resulted in seizure freedom. This study aims to call attention to the potentially curative surgical options for generalized tonic seizures and lends insights into the network pathophysiology of tonic seizures and LGS.

## 2 METHODS

This study received Institutional Review Board (IRB—P00045155) approval from Boston Children's Hospital (BCH). Given the retrospective study design, the IRB committee waived the requirement for informed consent. Five participants with generalized tonic seizures who underwent a surgical intervention (lesionectomy or LITT) for unifocal structural abnormalities between 2013 and 2024 were identified from the BCH epilepsy surgery database. These select cases represent neither a consecutive case series nor a comprehensive review of all patients operated for tonic seizures at our institution. Inclusion criteria were: (1) drug‐resistant epilepsy; (2) a focal structural abnormality evident on MRI; (3) generalized tonic seizures as the targeted seizure type (4); classic tonic seizure semiology (symmetric and gradual bilateral stiffening and extension of upper extremities) as per ILAE descriptions^21^; (5) abrupt and generalized seizure onset on EEG, with diffuse attenuation and overriding generalized fast activity (often preceded by a spike and wave); and (6) complete seizure freedom of >1 year after resective or ablative surgery.

## 3 RESULTS

**FIGURE 1:**
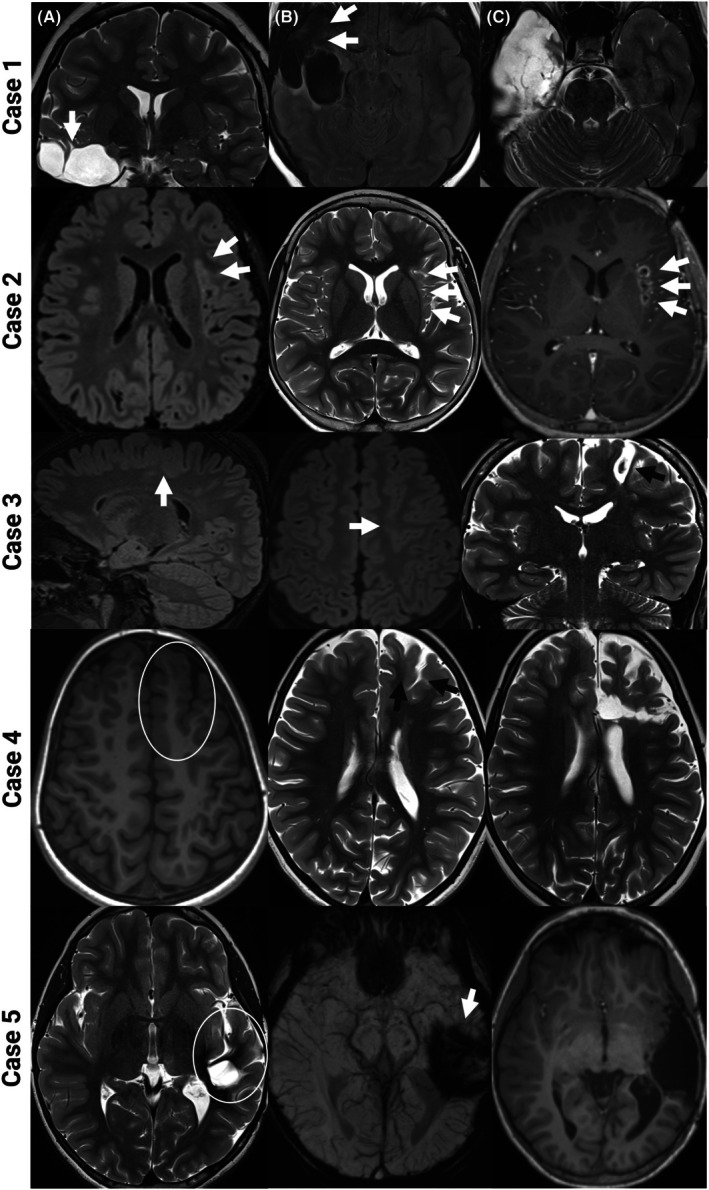
Preoperative and postoperative neuroimaging. Participant #1 (A) Coronal T2‐weighted image with prior resection cavity in right temporal lobe. Adjacent tissues are thickened, dysplastic, and have poor gray/white matter differentiation; (B) Axial FLAIR showing cystic encephalomalacia with T2‐hyperintense borders and residual dysplastic tissue anteriorly; (C) Postoperative axial T2‐weighted image after extensive temporal lobectomy. Participant #2 (A) Axial FLAIR with subtle T2 prolongation of anterior insula, and fuzzy border; (B) Axial T2‐weighted image shows thinning of the insula; (C) Axial T1 MPRAGE image with contrast, showing 3 discrete foci of laser interstitial thermal therapy (LITT) cavities (dark) with surrounding enhancement (light). Participant #3 (A) Sagittal FLAIR with deepened and poorly delineated sulcus, traveling into the white matter; (B) Axial FLAIR with hazy T2 hyperintensity; (C) Coronal T2 image shows the LITT cavity in the posterior superior left frontal lobe. Participant #4 (A) Axial T1 MPRAGE with frontal lobe deep cleft with surrounding cortical thickening; (B) Axial T2 shows the same, note poor gray/white matter boundary; (C) Axial T2‐weighted image showing disconnection of the anterior portion of the left frontal lobe. Participant #5 (A) Axial T2‐weighted image with resection cavity from prior cavernous malformation. Note T1 hypointense staining from hemosiderin; (B) Axial susceptibility‐weighted image (SWI) demonstrating extensive hemosiderin deposits far beyond the surgical cavity; (C) Axial T1 MPRAGE shows extension of the prior resection cavity.

All participants experienced their first seizure during childhood, with onset ages ranging from 1 month old to 7 years and 3 months old (Table 1). All but two participants had more than one seizure type. Interictal EEG features compatible with LGS were common, with three participants having generalized paroxysmal fast activity (GPFA) and two having generalized slow spike‐and‐wave (SSW) on EEG, of which two participants had both EEG patterns (Table 1). Most lesions were located in the frontal lobe (*n* = 3) (Table 2). All children were operated on in childhood, ranging from 5.6 to 9.5 years of age at the time of their first epilepsy surgery (Table 3). Three participants underwent a surgical resection (of which one had a concomitant frontal disconnection) and two ablative surgeries (LITT) (Figure 1). FCD, either suspected radiologically or confirmed via pathology, was the most common etiology (*n* = 3).

**TABLE 1:** Baseline characteristics of the study participants.

Participant	Seizure onset age	Preop ASMs	Seizure types	Seizure frequency	Interictal
1	3 years	LEV; TPM; LTG	Tonic	3/week	GPFA, SSW, focal spikes at P3, F7, intermittent slowing, epochs of electrodecrement
2	1 month (neonatal period)	VPA; LAC	Tonic	3/week	Left frontal and posterior temporal–parietal epileptiform patterns, irregular GSW, rare left frontal slowing
3	4 years, 6 months	LAC; LTG; CLB	Tonic, FIAS	1–5/day	GPFA, generalized polyspikes, multifocal spikes, left frontotemporal slowing, GSW
4	2 months	LEV; CLB; VPA; VGB	Tonic, spasms	3–5/day	Frequent midline and left frontal epileptiform discharges
5	7 years, 3 months	CBD; CLB; LAC; TPM	Tonic, spasms	3–4/day	GPFA/generalized spikes, SSW, 2–4 Hz left temporal slowing, frequent spikes left mid‐to‐posterior temporal lobe

Abbreviations: CBD, Cannabidiol; CLB, Clobazam; FIAS, Focal impaired awareness seizure; GPFA, generalized paroxysmal fast activity; GSW, generalized spike‐and‐wave; LAC, Lacosamide; LEV, Levetiracetam; LTG, Lamotrigine; SSW, Generalized slow spike‐and‐wave; TPM, Topiramate; VGB, Vigabatrin; VPA, Valproic acid.

**TABLE 2:** Preoperative characteristics of the study participants.

Participant	Lesion location	Handedness	Language involvement ipsilateral to lesion	MRI	PET	Etiology
1	Right temporal	Right	No (nonverbal at age 7, and nondominant temporal lobe)	Nonenhancing partially cystic, irregularly marginated area of T2 hyperintensity in the subcortical white matter of the right anterior/medial temporal lobe	Decreased tracer uptake right subcortical temporal lobe in area of lesion, normal adjacent cortical uptake	FCDIIIa/b (mesial temporal sclerosis, focal cortical dysplasia, low‐grade glial/glio‐neuronal neoplasm)
2	Left frontal	Left	No (based on fMRI)	Subtle thinning of the left posterior superior perisylvian cortex	Left insular/perisylvian and right posterior temporal hypometabolism	Vascular (perinatal infarct)
3	Left frontal	Right	Yes (based on fMRI)	Indistinct, elongated area of signal abnormality within the subcortical white matter of the left frontal lobe, which is somewhat nonspecific in appearance but may represent a subtle malformation of cortical development	Left precentral gyrus hypometabolism	Suspected FCD
4	Left frontal	Right	No (fMRI shows atypical right‐dominant language)	Nodular subependymal GMH along left lateral ventricle, and T2 prolongation in left superior frontal gyrus subcortical white matter associated with a frontal lobe deep cleft with surrounding cortical thickening consistent with malformation of cortical development	Subtle decreased FDG uptake in the anterior left frontal lobe	FCDIb (plus GMH)
5	Left temporal	Right	Yes (fMRI shows left lateralized expressive language and right lateralized receptive language with limited support from left)	Resection cavity from prior left temporal cavernous malformation	Left temporal hypometabolism	Vascular (resection cavity from prior cavernous malformation)

Abbreviation: GMH, gray matter heterotopia.

**TABLE 3:** Postoperative characteristics of the study participants.

Participant	Type of surgery	Age at epilepsy surgery (years)	Postoperative ASMs	Postoperative medication withdrawal (months)	Postoperative seizure outcome (Engel class, duration)	Postoperative change in development
1	Resection	7.0 (1st surgery); 16.8 (2nd surgery)	After 1st surgery, switched to OXC. After 2nd surgery, stayed on OXC	N/A	1st surgery: 1A, seizures returned as focal tonic after 8 years of seizure freedom 2nd surgery: 1A, 3 years follow‐up	Nonverbal at age 7 at time of 1st surgery. Postoperatively, acquired language at a developmental age equivalent to approximately 2–3 years old
2	LITT	7.0	VPA; LAC	N/A	1A, 2.7 years follow‐up	Parental report of improved cognition and language, but not formally tested
3	LITT	9.5	LAC; LTG	CLB (18)	1A, 4.4 years follow‐up	Preoperative deficits in expressive language (dysfluency), socialization and communication. Postoperative stable language but gradual improvement in other two domains
4	Frontal resection/disconnection	5.6	LEV; CLB; VPA	VGB (3)	1A, 1.7 years follow‐up	Developmentally appropriate (minor weaknesses in e.g., handwriting, emotional regulation and inattention). No significant change after surgery
5	Resection	8.8	LAC	CLB (6); CBD (12); TPM (18)	1A, 2.5 years follow‐up	No postoperative change in preoperative language processing (including dyslexia) and executive function deficits

Abbreviations: CBD, Cannabidiol; CLB, Clobazam; LAC, Lacosamide; LEV, Levetiracetam; LTG, Lamotrigine; OXC, Oxcarbazepine; TPM, Topiramate; VGB, Vigabatrin; VPA, Valproic acid.

Postoperatively, all participants were seizure free (Engel Class 1A) at follow‐up ranging between 1.7 and 4.4 years (Table 3). One participant was initially seizure‐free of generalized tonic seizures after resection of a right temporal mass (pathologically confirmed as FCDIIIa/b with FCD adjacent to low‐grade glial/glioneuronal neoplasm and mesial temporal sclerosis) for 8 years, after which seizures returned as focal tonic seizures. The second surgery extended the resection posteriorly and resulted in seizure freedom at the time of last follow‐up (3 years postoperatively). Three participants were able to wean at least one ASM during the postoperative stage.

### 3.1 Presentation of a selected participant (participant #3)

**FIGURE 2:**
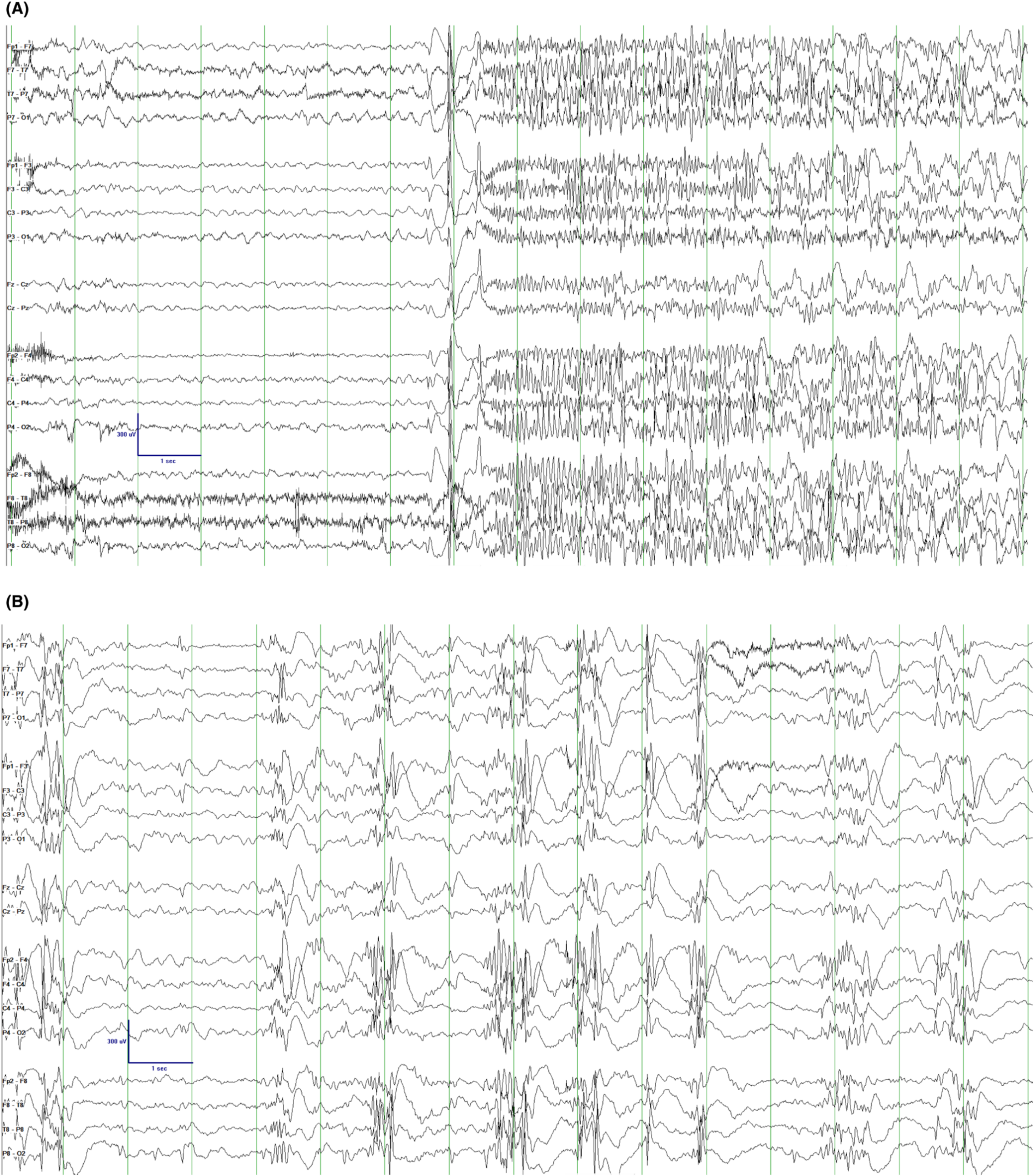
Participant #3. Generalized tonic seizures found on preoperative EEG, despite a small lesion in the left frontal lobe. This reinforces the concept of a “secondary network epilepsy.” LFF 1 Hz; HFF 70 Hz; Sensitivity 15 μV; Time base 30 mm/s (A). Preoperative EEG showing generalized fast polyspikes during sleep, similar to GPFA. LFF 1 Hz; HFF 70 Hz; Sensitivity 15 μV; Time base 30 mm/s (B). EEG, electroencephalography; HFF, high‐frequency filter; LFF, low‐frequency filter.

**FIGURE 3:**
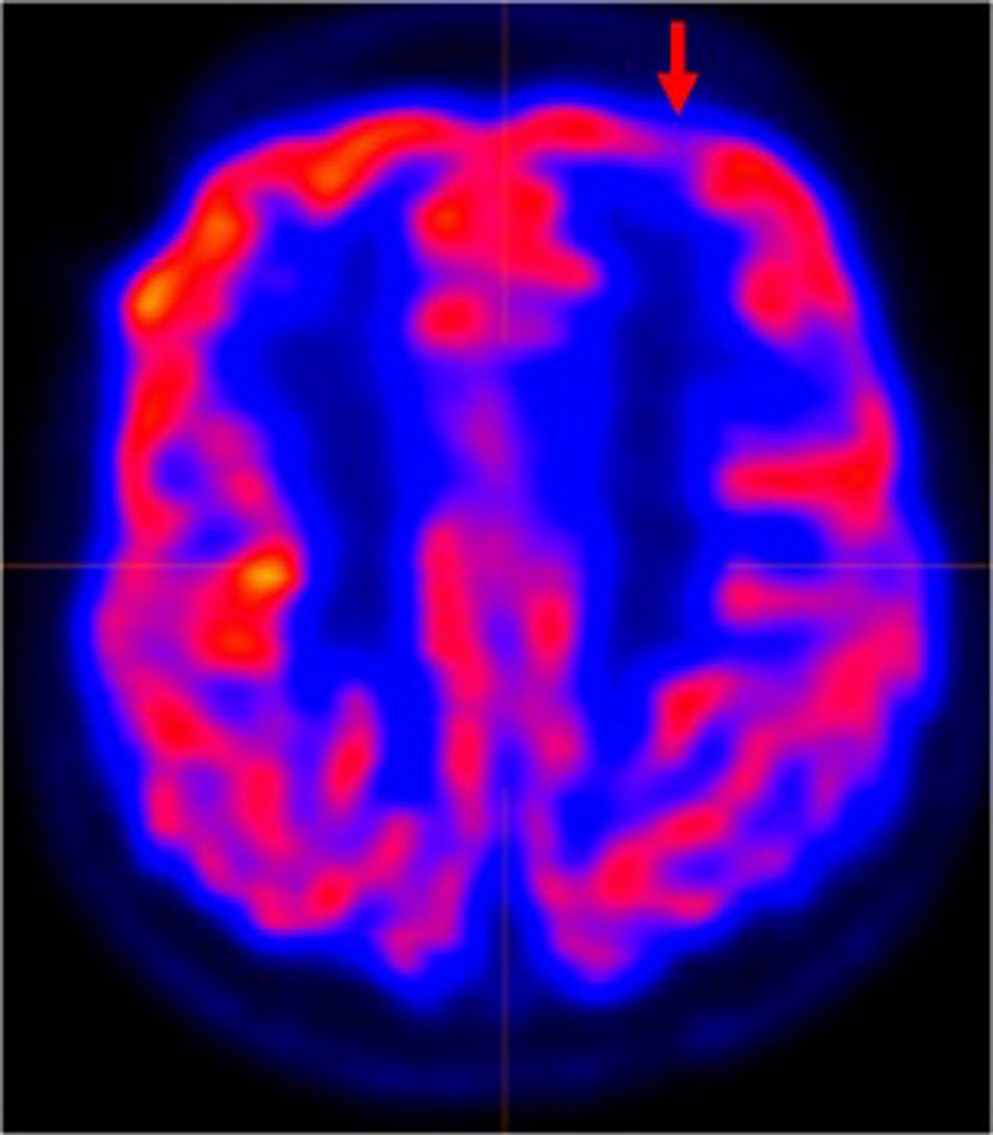
Participant #3. Preoperatively, left precentral gyrus hypometabolism was found on FDG‐PET (indicated by the red arrow). Cool, blue colors represent areas of lower metabolism, while hot, red colors signify higher metabolism.

At age 4 years, a developmentally normal male child developed seizures with behavioral arrest, rightward eye and head deviation, and impaired awareness. Over time, he developed apparent generalized tonic seizures but with ongoing focal features including asymmetric bilateral arm raising (right arm more than left), head and neck stiffening, and occasional rightward head and eye version. Seizures typically lasted for 5–10 s and occurred both during the day and night. Seizures were refractory to lacosamide, lamotrigine, and clobazam, and he was referred for epilepsy surgery evaluation at age nine. Gradually, his seizure severity worsened prior to surgical evaluation, with tonic seizures becoming progressively longer and occurring up to five times daily and requiring clonazepam rescue medication up to five times weekly. Interictal EEG findings included frequent, predominantly left‐hemispheric multifocal sharp waves and bursts of sleep‐potentiated rapid generalized polyspikes, similar to GPFA,^22^ followed by predominantly left frontotemporal slowing (Figure 2B). The ictal EEG showed an archetypical tonic seizure pattern consisting of a high‐amplitude generalized spike followed by diffuse attenuation and bilateral low‐voltage fast activity, without obvious localizing features (Figure 2A). Structural MRI revealed an elongated area of signal abnormality within the subcortical white matter of the left frontal lobe, suspected to be FCD (Figure 1), and there was left precentral gyrus hypometabolism on FDG‐PET (Figure 3). Preoperatively, he received speech therapy for dysfluency and his overall intelligence was in the low average range. Other limitations were found in daily living, socialization, and communication skills.

**FIGURE 4:**
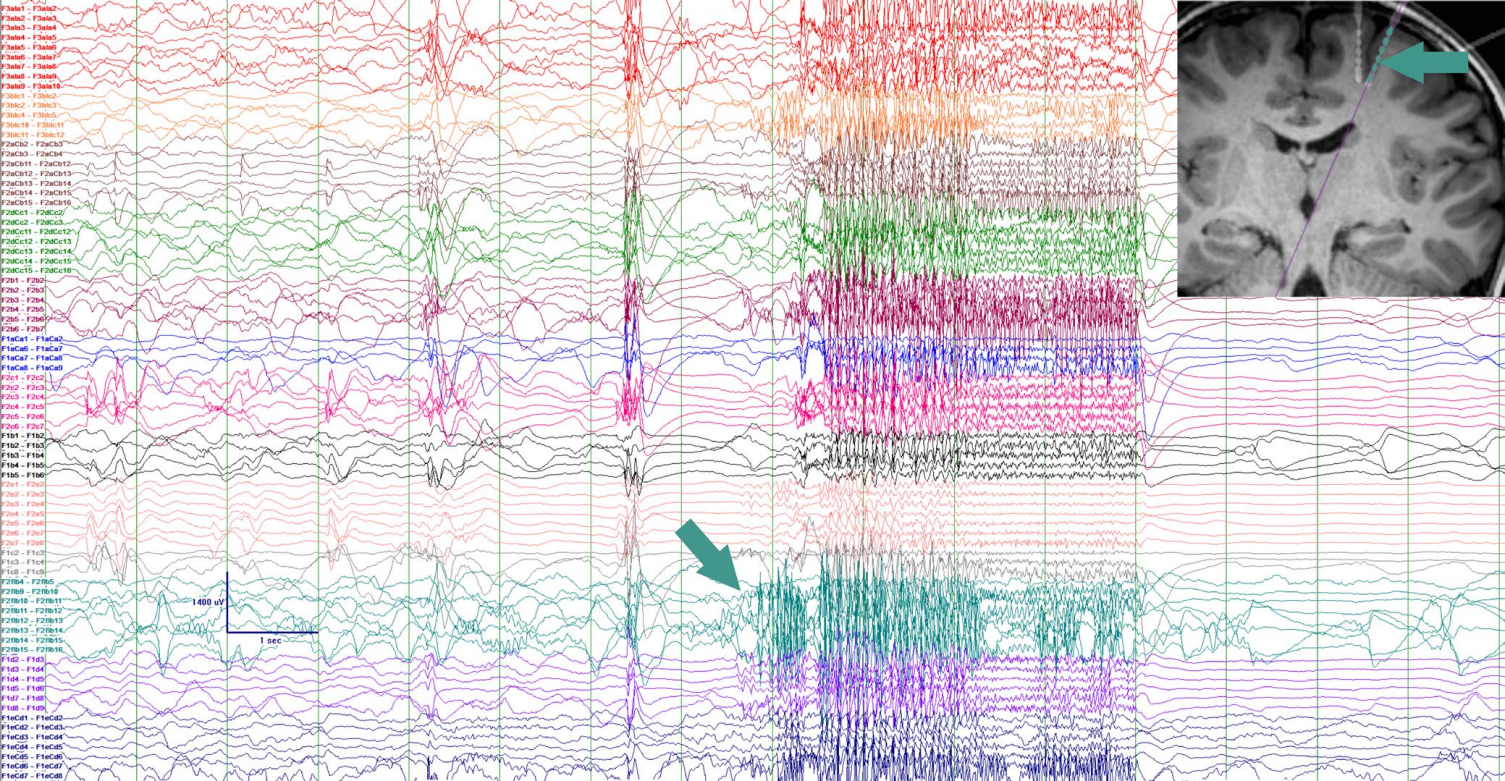
Participant #3. sEEG revealed a seizure onset zone involving multiple left frontal electrodes, with the most prominent involved channels here highlighted for illustration purposes with the forest green arrow. In the top‐right corner of the image, the forest green arrow indicates the corresponding location of the electrode on the coronal T1‐weighted MRI. sEEG, stereoelectroencephalography.

For concerns of broad interictal discharges, to discriminate focal onset with rapid propagation from generalized seizure onset, and to allow for stimulation of eloquent (motor) cortex, the conference reached consensus for left frontotemporal stereoelectroencephalography (sEEG) implantation. On sEEG, the seizure onset zone was broad and involved multiple left frontal electrodes (Figure 4). The earliest ictal patterns localized to the posterior left middle frontal gyrus and the superior frontal sulcus. After multidisciplinary review, due to the presence of an MRI visible lesion in proximity to the motor cortex, magnetic resonance thermography‐guided LITT was offered. The participant underwent the procedure without complications. He had two seizures in the days following his operation, and since then, he has been entirely seizure‐free from all seizure types, with the last follow‐up at more than 4 years postsurgery. An interictal EEG at 9 months postoperatively showed focal left temporoparietal spikes and left‐predominant generalized discharges. He was able to wean clobazam at 18 months postsurgery, though lamotrigine and lacosamide were not withdrawn. Postoperatively, he was diagnosed with ADHD. Behavioral concerns declined markedly per parental report at the time of clinic visits. He continued with speech therapy at school but graduated from other therapies. Teacher reports mention that he is doing well socially and academically, with minimal support and increased participation in class.

## 4 DISCUSSION

This study demonstrates that focal cortical lesions can drive generalized EEG and seizure patterns, including tonic seizures, and that lesion ablation or removal can lead to sustained seizure freedom. The idea that generalized seizures could stem from a small cortical lesion has traditionally been met with doubt. Some authors postulated that generalized seizures begin in the thalamus and brainstem,^23^ with subsequent spread to the cortex. Recently, the network effects of focal lesions have become more apparent. For patients with LGS specifically, a study of simultaneous scalp EEG with thalamic depth electrode recordings in patients undergoing DBS surgery found the thalamus to consistently lag the cortex by ~100 ms during generalized interictal discharges, particularly GPFA.^24^ In the current literature, the benefits of LITT ablations have mostly been described in cases of medically refractory focal epilepsy.^25^, ^26^ Here, we show that seizure freedom with LITT ablation of focal lesions can also be achieved in cases of generalized tonic seizures.

The concept of “secondary network” epilepsy may help to explain how focal lesions can drive generalized EEG findings and tonic seizures.^6^, ^8^ This framework refers to the hypothesis that diverse etiologies converge on similar brain networks, thereby giving rise to shared electroclinical features. For example, studies using simultaneous EEG with functional MRI (EEG‐fMRI) in patients with LGS have shown that bursts of GPFA are expressed via a consistent brain network involving the frontoparietal cortex, thalamus, brainstem, and cerebellum.^3^ This network is similar across patients with focal lesions in different cortical locations, and in patients with various genetic or other causes, suggesting the pathological network activity develops “secondary” to the initiating epileptogenic insult, likely via complex interactions between ongoing neurodevelopmental processes, the underlying etiology, and recurrent epileptic activity during critical maturational periods. Over time, this network may become autonomously capable of sustaining epileptic activity even independent of the original etiology, perhaps contributing to the decreasing efficacy of treatments like lesionectomy with greater delays between seizure onset and surgery.^9^, ^27^

This emphasizes the importance of early surgical referral in patients with tonic seizures in whom a focal cause is confirmed or suspected. Removal of the lesion, by eliminating the destabilizing seizure focus, may halt the evolution of a secondary epileptic network and allow the return of normal brain network maturation and cognitive development.^28^ This is supported by studies comparing presurgical and postsurgical fMRI scans in children with LGS.^11^ Seizure freedom is associated with restoration of fMRI connectivity patterns that more closely resemble those of a healthy brain, including stronger within‐network integration and between‐network segregation, alongside improvements in behavior and developmental skills.^11^, ^29^

In addition, the concept of focal lesions causing generalized epilepsy by engaging a secondary network might account for the so‐called “running‐down” or “winding‐down” phenomenon.^8^ Though rarely seen clinically and difficult to recognize retrospectively, this phenomenon describes the gradual remittance of seizures and interictal epileptiform discharges in the weeks or months following surgery and has been occasionally reported in both focal and generalized epilepsies and LGS specifically.^8^, ^30^, ^31^ This might reflect a gradual return of normal connectivity and excitatory‐inhibitory balance in vulnerable brain areas remote from the lesion. However, the precise limits of this “window of reversibility” remain unclear—that is, the time after seizure onset when the secondary network is no longer dependent on the lesion, at which point surgery may become less effective at stopping seizures or improving cognitive outcomes. Indeed, previous studies have demonstrated inferior seizure outcomes when durations between epilepsy onset and epilepsy surgery are longer, suggesting expansion of a secondary epilepsy network despite structurally static lesions.^32^, ^33^ Further, while here we demonstrate resolution of generalized tonic seizures caused by focal lesions, it is important to realize that these cases represent only a subset of children with focal lesions causal of an epileptic encephalopathy; mere presence of a surgically remediable focal lesion does not guarantee sustained seizure freedom or reversibility of the full electroclinical spectrum of LGS.

The knowledge gap in the timeline of an emerging epileptic encephalopathy and the effects of treatment timing warrant further investigations into network architecture of tonic seizures and electrophysiological biomarkers of treatment response in LGS. In a previous study in adults with LGS undergoing DBS of the thalamic centromedian nucleus, GPFA burden from 24‐h EEG recordings correlated with the magnitude of seizure reduction over the following 3 months.^34^ This suggests that interictal EEG may serve as a biomarker of secondary network dysfunction and could be used to guide rapid therapy titration. Lesion network mapping (LNM),^35^ a technique that examines how brain lesions lead to shared neurological phenotypes through common connectivity patterns, may provide further insights into the brain areas most vulnerable to the development of tonic seizures and LGS.^36^ The same underlying pathology (e.g., FCD) can manifest as LGS, focal epilepsy, or other phenotypes, implying that lesion location (and thus the specific connected circuits) may influence clinical presentation. With a sufficiently large patient cohort, including individuals with and without tonic seizures, LNM could help pinpoint specific cortical “hotspots” that are most likely to trigger this seizure type. This could improve predictions of tonic seizure risk in lesional cases, enabling preventative interventions or refining targets for neuromodulation in patients without lesions.

## FUNDING INFORMATION

Support for the Localization Laboratory came from the Son‐Cundy family, the Strem family, the MacPherson Fund, Inc., and a generous contribution from Dr. Susanna A Hayes. AELW was funded by the LGS Foundation and the Pediatric Epilepsy Research Foundation.

## CONFLICT OF INTEREST STATEMENT

None of the authors have any conflict of interest to disclose.

**Test yourself**

1. Which feature is NOT required for the diagnosis of Lennox–Gastaut syndrome according to the latest ILAE classification?
  - Tonic seizure
  - SSW and GPFA on EEG (or history of these findings on EEG)
  - MRI abnormalities
  - Age at onset <18 years
2. What is the definition of drug‐resistant epilepsy, based on a 2010 consensus proposal by an ILAE Task Force (see citation below)?
  “Drug resistant epilepsy may be defined as failure of adequate trials of ______ tolerated and appropriately chosen and used AED schedules (whether as monotherapies or in combination) to achieve sustained seizure freedom.”
  (Kwan P, Arzimanoglou A, Berg AT, Brodie MJ, Allen Hauser W, Mathern G, Moshé SL, Perucca E, Wiebe S, French J. Definition of drug resistant epilepsy: consensus proposal by the ad hoc Task Force of the ILAE Commission on Therapeutic Strategies. *Epilepsia*. 2010;51(6):1069–77. http://doi.org/10.1111/j.1528‐1167.2009.02397.x. Epub 2009 Nov 3. Erratum in: *Epilepsia*. 2010;51(9):1922.)
  - Two
  - Three
  - Four
3. In a recent consensus article, the Surgical Therapies Commission of the ILAE recommended referral for surgical evaluation for every patient with drug‐resistant epilepsy below which age (see citation below)?
  (Jehi L, Jette N, Kwon CS, Josephson CB, Burneo JG, Cendes F, Sperling MR, Baxendale S, Busch RM, Triki CC, Cross JH, Ekstein D, Englot DJ, Luan G, Palmini A, Rios L, Wang X, Roessler K, Rydenhag B, Ramantani G, Schuele S, Wilmshurst JM, Wilson S, Wiebe S. Timing of referral to evaluate for epilepsy surgery: Expert Consensus Recommendations from the Surgical Therapies Commission of the International League Against Epilepsy. *Epilepsia*. 2022;63(10):2491–2506. http://doi.org/10.1111/epi.17350. Epub 2022 Jul 17.)
  - 60 years or younger
  - 70 years or younger
  - 80 years or younger

*Answers may be found in the* *supporting information*

## Supporting information


Data S1.

## Data Availability

The data that support the findings of this study are available on request from the corresponding author. The data are not publicly available due to privacy or ethical restrictions.
